# An iterative design process to develop a randomized feasibility study and inform recruitment of minority women after stillbirth

**DOI:** 10.1186/s40814-019-0526-2

**Published:** 2019-11-27

**Authors:** Jennifer Huberty, Jeni Green, Katherine J. Gold, Jenn Leiferman, Joanne Cacciatore

**Affiliations:** 1 College of Health Solutions, Arizona State University, 500 N. 3rd St., Phoenix, AZ 85004 USA; 2 Department of Family Medicine, Department of Obstetrics & Gynecology, University of Michigan, 1018 Fuller Street, Ann Arbor, MI 48104-1213 USA; 3 Colorado School of Public Health, University of Colorado Denver, 13001 E. 17th Place, B119 Bldg 500, Room E3341, Anschutz Medical Campus, Aurora, CO 80045 USA; 4 School of Social Work, Arizona State University, 411 N. Central 8th Floor, Phoenix, AZ 85004 USA

## Abstract

**Background:**

Yearly, approximately 25,000 US women experience stillbirth and African American women have a 2.2 fold increased risk of stillbirth compared with white women. After stillbirth, women are subject to a sevenfold increased risk of post-traumatic stress compared with women after a live-birth. This paper presents findings from phase one of a National Institutes of Health funded, two-phase feasibility study to examine an online yoga intervention to reduce symptoms of post-traumatic stress in mothers after stillbirth. An iterative design was used to (1) inform the development of the online yoga intervention and (2) inform recruitment strategies to enroll minority women into phase two.

**Methods:**

Ten mothers (*N* = 5 stillbirth moms with no yoga experience, *N* = 5 nonstillbirth moms with yoga experience) participated in a series of online yoga videos (*N* = 30) and were assessed for self-compassion (SC) and emotional regulation (ER) before and after each video. An independent group of five minority women who had experienced stillbirth were interviewed about cultural barriers to recruitment and perceptions/opinions of yoga. A mean was calculated for SC and ER scores for each video at pre- and post-time points. The percent change of the mean difference between pre-post SC and ER scores were used to select videos for phase two. Videos with a negative change score or that had a 0% change on SC or ER were not used. A combination of deductive and inductive coding was used to organize the interview data, generate categories, and develop themes.

**Results:**

Five of the 30 tested yoga videos were not used. An additional 12 videos were developed, filmed, and used in the prescription for phase two. Topics from interview findings included perceived benefits/barriers of and interest in yoga, preferred yoga environment, suggested recruitment methods, content of recruitment material, and recommended incentives.

**Conclusions:**

Online yoga may be beneficial for improving emotional regulation and self-compassion, but further testing is needed. Additionally, minority women express interest in online yoga but suggest that researchers apply culturally specific strategies regarding methods, content of material, and incentives to recruit minority women into a study.

## Introduction

Stillbirth is devastating and life changing [1] and has a significant emotional and mental health impact on women and their families. In the USA, it is estimated that 25,000 women annually experience the death of a baby to stillbirth (in-utero fetal death at ≥ 20 weeks gestation) [2, 3]. The risk of stillbirth is greater for ethnic minorities with African American women having a 2.2 fold increased risk of stillbirth compared with white women [4]. When compared with women who deliver a live baby, women who deliver a stillborn baby have a nearly sevenfold increased risk of post-traumatic stress (PTS), fourfold increased risk of depressive symptoms, and twofold increased risk of anxiety symptoms [5–7]. Approximately 44% of mothers who have stillborn babies exhibit symptoms of post-traumatic stress, which may last anywhere from 2 months to 18 years [8–11].

Recently, stillbirth has been recognized as a major and neglected global health burden [12, 13]. In 2011 and 2016, The Lancet released two series dedicated entirely to stillbirth [14, 15], highlighting its causes and prevention strategies and issuing a global call for action. Though the silence surrounding stillbirth is slowly being broken, there is a considerable amount of work to be done especially regarding mental and emotional health. Current treatment models, particularly related to post-traumatic stress, are lacking. The most common treatments for post-traumatic stress after stillbirth are support groups/counseling and/or psychiatric medication [16–19].

While these strategies have been shown to be effective in other populations with post-traumatic stress, they may not be sufficient for bereaved mothers. Many mothers express a strong interest in home-based treatments as they do not want to encounter other babies in public. Support groups may not be effective for post-traumatic stress as some women may be reluctant to communicate with others [20, 21]. Additionally, many grieving mothers decry the use of psychiatric medications [22], as many are interested in conceiving again (50–98% conceive again) [22–24]. In a 2016 international survey, of 2716 parents, 66% conceived their subsequent child in the first 12-months after their baby’s death [25].

A recent systematic review evaluating intervention studies that target mental and/or physical health outcomes in women who have experienced stillbirth found only two intervention studies that aimed to improve mental health and none for physical health [26]. There is a need for novel and effective approaches to address post-traumatic stress in bereaved mothers that consider the unique needs of this population. Yoga may be a feasible, effective treatment for reducing symptoms of post-traumatic stress in women who have experienced stillbirth [27]. There is evidence for the effects of yoga in improving mental health in other populations including pregnant and post-partum women [28].

**Fig. 1:**
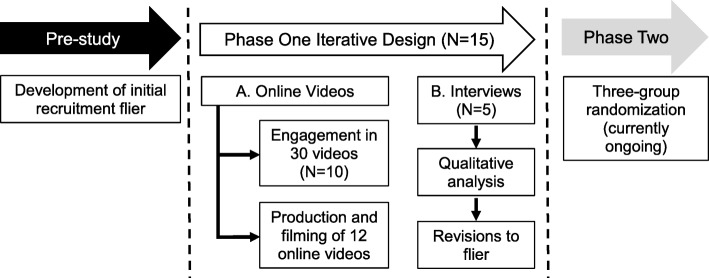
Study flow

The purpose of this paper is to present findings from phase one of a two-phase, three-group randomized feasibility study funded by the National Institutes of Health (NIH# R34AT008808). The main objective of the two-phase study is to determine the feasibility of an online yoga intervention to reduce symptoms of post-traumatic stress (PTS) in mothers who have experienced the death of a baby to stillbirth (currently ongoing). Phase one used an iterative design to (1) inform the development of the online yoga intervention (phase two, currently in progress) and (2) inform recruitment strategies to enroll minority women into phase two and future studies. Phase one data collection and how the information was used to implement phase two is described below. See Fig. 1 for study flow.

## Methods

The Institutional Review Board of a large university in the Southwestern United States approved this study. Informed consent was obtained from all individual participants included in the study. To inform the development of the yoga intervention for phase two, mothers (*N* = 10) were asked to view and engage (i.e., participate) in a series of online yoga videos while being observed by the research team. Women were then assessed for emotional regulation and self-compassion before and after completing each video. To inform recruitment strategies, a separate and independent group of bereaved minority women (*N* = 5) who had experienced stillbirth were asked to participate in a brief phone interview. Women were asked to identify and share cultural barriers to recruitment and perceptions/opinions of yoga to inform future development of an online yoga prescription (phase two). The entire study design (including both phase one and two) has been described elsewhere in detail [29].

### Participant recruitment and selection

Participant recruitment was the same for both the engagement of the online videos and the interviews. Research staff contacted stillbirth-related non-profit organizations (e.g., Stories of Babies Born Still, Star Legacy Foundation) and asked them to advertise the study by posting provided recruitment information (e.g., flyers, blurbs) to their social media sites, websites, newsletters, and/or listservs. Participants were also recruited via Facebook and Instagram.

Women who were recruited for the engagement in the yoga videos responded to an advertisement that included messaging for women to “try yoga” (via online yoga videos) and provide feedback. Women who were recruited for the interviews responded to an advertisement specifically requesting participation from African American and/or Hispanic women who had experienced stillbirth to share their perceptions about yoga. Recruitment for this study occurred between September 2016 and June 2017. None of the women in phase one were exposed to an intervention.

### Eligibility

Eligibility criteria for the engagement in online videos and interviews are listed in Table 1. Engagement of online videos included two distinct groups of non-pregnant women: (a) women (*N* = 5) who had experienced a stillbirth (within 6 weeks–32 months) and did not regularly practice yoga (< 60 min/week), and (b) women (*N* = 5) who did not have a previous stillbirth history but had delivered at least one live-born infant and regularly practiced yoga (≥ 60 min/week). We recruited stillbirth moms without regular yoga practice and non-stillbirth moms with regular yoga practice to make sure we had different perspectives from women with different experiences.

**Table 1:** Eligibility criteria for phase one

Engagement in online videos	Interviews
Inclusion	
• Able to read/understand/speak English	• Able to read/understand/speak English
• Women who experienced stillbirth within past 6 weeks–32 months who do not regularly practice yoga OR	• Racial/ethnic minority women
	• Women who experienced stillbirth within past 6wks-24 mos
• Women who have NOT experienced stillbirth but have given at least one live birth and regularly practice yoga	• Do not regularly practice yoga
	• Underactive (≤120 mins/wk moderate intensity physical activity)
Exclusion	
• Pregnant women	• Caucasian women
• Unstable psychiatric condition (psychosis; suicidal ideation with plan)	• Unstable psychiatric condition (psychosis; suicidal ideation with plan)

Regular yoga practice defined as > 60 min/week; stillbirth defined as in-utero fetal death at ≥ 20 weeks gestation

Eligibility for the interviews included self-identified Black, African American, or Native African and/or Hispanic women (*N* = 5) who had experienced stillbirth (within 6 weeks–32 months) and did not regularly practice yoga. The women who participated in the interviews were independent from those who engaged in the videos.

## Procedures

### Engagement in videos

Women who were interested in participating with the engagement of the yoga online videos were asked to complete an eligibility screener via Qualtrics, a web-based survey software (Provo, Utah). Ineligible women were notified of their status via email. Eligible women were asked to schedule an in-person appointment to sign an informed consent and complete their first video. An experienced, masters trained, yoga biomechanist and educator, and PhD level senior investigator/yoga instructor identified 30 beginner/intermediate yoga videos from the Udaya (i.e., fee-based online streaming yoga website) library for review (over 800 total videos in library). Women engaged in the online yoga videos for free. Selection criteria for the 30 yoga videos included the following: (1) videos that were used in our previous beta-testing [27] and were positively reviewed by mothers who experienced a stillbirth, (2) classes that were slower moving (i.e., beginner-based) with detailed instruction and alignment cues (i.e., safe and proper form), (3) classes that were safe for women up to 20-week gestation in the case a participant became pregnant during the intervention, and (4) videos that the research team believed would cultivate emotional regulation and self-compassion. For example, the research team considered evidence-based proposed mediators from a general population with post-traumatic stress, tone of the teacher (i.e., calming, accepting), commentary during the video (i.e., self-love, acceptance, being aware of emotions), and poses (e.g., forward fold, child’s pose, heart openers).

The 30 yoga videos were randomized so that each video was reviewed three times by three different women. Each woman was asked to review nine yoga videos (20–30 min in length) and were limited to review only one video per day. They were asked to participate in the yoga videos by themselves in an observation room at a university in the Southwestern USA. The research team set up a yoga mat, two blocks, and a laptop computer with the woman’s video for the day and observed her outside of the room (to ensure she was engaging in the video). If women were unable to schedule an appointment to review the video at the lab, the women were able to review the video at home while a research staff observed the session via web-conferencing (i.e., Skype, FaceTime). Before the start and at the end of participation in each of the nine videos, women were asked to complete two, 1-item (not validated) investigator developed 5-point Likert-scales assessing emotional regulation (ER) and self-compassion (SC). The questions are listed in Table 2. The two, 1-item questionnaires for ER and SC were created to reduce participant burden that may have occurred by completing a validated measure for ER (10 items [30]; or SC (26 items [31];) before and after engaging in a yoga video. The scores obtained from each video (*N* = 3) were averaged to determine a meaningful change in ER and/or SC. Videos producing an increase in ER and/or SC were integrated into the prescription for phase two.

**Table 2:** Pre and post self-compassion and emotion regulation assessments

Questions	Answer choices
1. How much self-compassion do you feel (i.e., self-kindness, understanding toward yourself and your grief, common humanity, mindfulness) right now?	a. Not much b. Little c. Somewhat d. Much e. A great deal
2. How much do you feel that you are able to be with your emotions (i.e., consciously stay with your emotions or mood) right now?	

In addition to having women engage with the online yoga videos, the research team filmed 12 additional, 30-min yoga videos in partnership with Udaya. The videos were filmed and developed prior to phase one to be integrated into the prescription for phase two, along with the tested existing Udaya videos. The design of the newly filmed videos was based on feedback from a previous beta-test of an online yoga intervention in women after stillbirth [27] and was approved by the MS-trained yoga biomechanist and educator and the study team. These videos were filmed specific for the current study population whereas existing Udaya videos are designed for the general population. For the control group, the research team filmed eight, 30-min stretching/toning videos (based on McAuley’s evidence-based stretching, limbering, and toning control groups for older adults) [32–36].

### Interviews

Women who were interested in participating in the interviews were asked to complete an eligibility screener via Qualtrics, a web-based survey software (Provo, Utah). Ineligible women were notified of their status via email. The first five eligible women were sent an informed consent via Qualtrics and asked to schedule an appointment with a trained research staff to complete a 15–20 min phone interview. Participants were compensated with a $15 gift card and one free month of online streaming yoga services on Udaya. A semi-structured, 17-item investigator-developed interview guide was used to ask questions related to (1) experiences and perceptions about yoga and (2) strategies to ensure adequate enrollment and retention of non-Caucasian women to participate in an online yoga intervention. Example questions from the interviews are listed in Table 3. Modifications were made to the recruitment flier based on recommendations identified from the interviews (See Fig. 3 for the revisions to the flier).

**Table 3:** Example interview questions

No.	Question	Prompts
Perceptions of yoga		
1	Do you have any family members/friends that participate in yoga?	a. If so, have they shared with you their perceptions of yoga? b. How has this impacted your perception?
2	Mindfulness can be defined as “the quality or state of being mindful or aware”. What do you think of utilizing complementary approaches like yoga or mindfulness to cope with the death of your baby?	
3	What do you think of participating in yoga at home, online (logging onto a yoga website) as compared with a group-based setting?	a. Would you prefer to do yoga in your home or at a yoga studio? Why? b. What type of an environment for yoga participation would be most useful after the loss of your baby? (examples: online, yoga studio, hospital-based setting, rec center)
4	What are some of the things that might get in the way of you beginning your own yoga practice?	
Recruitment strategies		
5	Much of the research has focused on participation in yoga for Caucasian women. Do you think that [IDENTIFIED RACE] women who have experienced the death of a baby to stillbirth would be interested in an online yoga study?	a. Why or why not?
6	When do you think the best time would be—how long after the death of the baby should we recruit grieving mothers for the study?	
7	What would an ideal social media post look like to you?	a. Is there any language that we should avoid using? b. Is there any language that you encourage us to use?
8	In your opinion, what would be the best way to let other grieving mothers from diverse backgrounds know about the study?	
9	If you were to participate in a study such as this, what would help motivate you to enroll in, continue, and eventually finish the study (i.e., online yoga) over the 12 weeks.	

### Data analysis

Data from the engagement in the yoga videos were entered into the Statistical Package for Social Sciences (SPSS) version 23.0 for analysis. Descriptive statistics (mean + SD, frequencies, and percentages) were used to describe the demographics of study participants. A mean was calculated for SC and ER scores for each video at pre- and post-time points. The percent change of the mean difference between pre-post SC and ER scores were used to select videos for the phase two yoga prescription.

Interview transcripts were imported into NVivo 12 for coding. A combination of deductive and inductive coding was used to organize the data, generate categories, and develop themes in a hybrid coding approach widely used in thematic analysis [37]. In this approach, top-level themes were identified based on the main interview questions. Sub-themes that emerged from the data also reflected the lived experiences and personal views of the research participants. Several stages of coding were used in which the overall distribution of research data by themes was continually reviewed and revised until the final definitions of themes and codes most accurately reflected the data. Two researchers separately coded the whole body of data in this way, and the final process of analysis drew on both coded datasets in order to improve the overall rigor of the study [38].

## Results

**Fig. 2:**
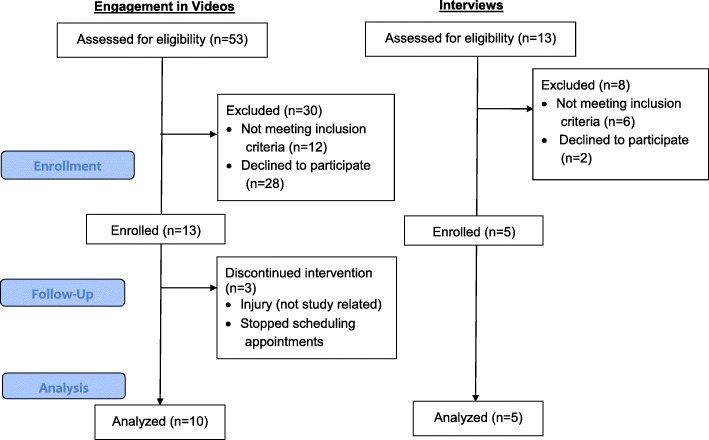
Participant flow into the study

Figure 2 illustrates the participant flow into the study for both participants that engaged with the videos and those who participated in the interviews. Participant demographics are listed in Table 4. Women who participated in the engagement of the yoga videos were on average 36 years old and primarily White, European-American, or Caucasian (90%; *N* = 9). Women participating in the interviews were on average 35 years old with 60% (*N* = 3) identifying as Black, African American, or Native African and 40% (*N* = 2) Hispanic. Race categories are not mutually exclusive (e.g., answer selections included check all that apply).

**Table 4:** Phase one participant demographics (*N* = 15)

Variable	Engagement in videos (*N* = 10)		Interviews (*N* = 5)	
Age M ± SD (years)	36	± 4	35	± 5
Race	*N*	%	*N*	%
White, European-American, or Caucasian	9	(90)	-	-
Black, African-American, or Native African	1	(10)	3	(60)
Native Caribbean or Afro-Caribbean Islander	-	-	1	(20)
Bi-Racial or Multi-racial	1	(10)	-	-
Other	-	-	2	(40)
Ethnicity				
Hispanic	2	(20)	2	(40)
Non-Hispanic	8	(80)	3	(60)

Race categories are not mutually exclusive. Answer selections are “check all that apply”

### Engagement (i.e., participation) in videos

Table 5 outlines the self-compassion (SC) and emotional regulation (ER) pre-post change scores. Videos with a negative change score or that had a 0% change on SC or ER were not used as part of the yoga prescription in phase two (*N* = 5). The remaining yoga videos were arranged in a sequence for the 12-week intervention (*N* = 25 total videos).

**Table 5:** Self-compassion (SC) and emotional regulation (ER) pre-post change scores

Video name	Minutes	Difficulty	SC ∆	SC % change	ER ∆	ER % change
Cultivate Strength	60	1	1.33	44.4%	1.00	33.3%
Inspire your life- Gratitude	60	1	1.00	30.0%	1.33	44.4%
Beach Meditation	15	1	1.00	33.3%	1.33	44.4%
Yoga foundations 1	60	1	1.00	30.0%	1.67	55.6%
All Levels Flow	60	1	1.00	33.3%	1.00	33.3%
Cultivate Love	60	1	1.00	30.0%	1.33	40.0%
Detox and Destress	60	1	1.00	30.0%	1.00	30.0%
Post Game 1 Hip Freedom	60	2	1.00	33.3%	1.00	33.3%
Rise and Shine: Quick Morning Flow	30	2	0.67	22.2%	0.67	22.2%
Experiment With Your Comfort Zone	20	1	0.67	22.2%	0.67	22.2%
Beginner Friendly Flow	40	1	0.67	20.0%	0.67	22.2%
Heart Opening	60	2	0.67	18.1%	1.00	30.0%
Tadasana	30	1	0.67	18.1%	0.67	20.0%
Cultivate Balance	60	1	0.33	11.1%	1.00	42.9%
Joyful	30	1	0.33	10.0%	1.00	33.3%
Harp Meditation	20	1	0.33	11.1%	0.67	22.2%
Contemplating Contentment	30	2	0.33	10.0%	0.33	10.0%
Get Grounded	20	1	1.00	33.3%	0.33	9.1%
Inspire your life- Creativity and Play	60	1	0.33	9.0%	1.00	30.0%
Get Fluid	20	1	0.33	8.3%	1.00	33.3%
Cultivate Courage	60	1	0.67	22.2%	0.33	9.1%
Yoga Chill Lounge	40	1	0.67	20.0%	0.33	9.1%
Get Grounded	40	1	0.67	18.2%	0.33	9.1%
Gentle Yoga	60	1	0.33	7.7%	0.67	16.7%
Warrior Free Flow	20	1	0.33	8.3%	0.33	8.3%
Make Time to Slow Down^a^	20	1	0.67	20.0%	− 0.33	− 10.0%
Unfolding the Lotus^a^	60	2	0.00	0.0%	0.33	10.0%
Love Thyself^a^	60	1	0.00	0.0%	1.00	37.5%
Mountain Warrior^a^	30	1	0.67	18.2%	0.00	0.0%
Stress Rescue^a^	20	1	0.67	18.2%	0.00	0.0%

Difficultly 1 = beginner, 2 = intermediate; ^a^Videos were not used for final prescription

### Filming of additional yoga videos

As mentioned above, based on feedback from a beta-test of an online yoga intervention in women who had experienced stillbirth, we also developed and filmed yoga videos using expertise from the yoga biomechanist/educator and the PhD trained researcher. Two trained yoga instructors (Caucasian female, African American male) were asked to develop sequences and language for 12 videos specifically for women who had experienced a stillbirth. The research team reviewed the sequences and language, provided feedback and modifications prior to filming, and produced the videos in partnership with Udaya.com. These videos were added to the sequence developed from the Udaya library (see above) and were not reviewed by study participants.

### Interview findings about perceptions of yoga and recruitment of minority women

#### Perceived benefits of yoga for women after a stillbirth

All five women (no regular yoga practice) acknowledged that yoga might be helpful in various ways, including easing physical discomfort or pain (one woman had been experienced pain since giving birth) and helping to prepare the body physically for a future healthy pregnancy. Mental or psychological benefits were mentioned by four of the participants and were conceptualized in terms of helping them to cope with the sadness of their loss by staying more present in the moment or practicing gratitude, for example:

*Not to make me forget but to help me like not constantly think about it every moment of the day and to be present and to be thankful for what is in front of me at the moment (Interview 4).*

#### Perceived barriers to yoga participation

Three main barriers to yoga participation were identified: cost, time constraints, and lack of energy. One of the participants remarked that “the cost of yoga classes here is expensive” (interview 4), and a second explained that since she has been mainly unemployed since her pregnancy, she cannot afford such additional costs (interview 5). Another of the participants reported that time rather than money is the most significant barrier to yoga participation, since she has a young child, a part time job, and household responsibilities, all of which constrain her available time. This participant also cited lack of energy as a factor hindering her participation in yoga (interview 3).

#### Perceived interest in yoga

When asked for perceptions of whether women of the same ethnicity as themselves who had also experienced a stillbirth would be interested in participating in an online yoga study, one of the participants (Hispanic, interview 2) expressed that other Hispanic women in this situation would be interested in an online yoga study. She also stressed that she felt any woman with this experience would be interested, regardless of ethnicity:

*I think is good not only for the Hispanic women or the African women I think it is good for all women in the same situation because it is something very sad and … it is difficult … for every women … to go on … (interview 2)*

The other participants indicated that other women in their ethnic group may not be likely to have a high level of interest in participating in such a program. The main reasons cited were cost/time factors and cultural factors. One of the participants observed that yoga is not generally popular among the African American population, with its members tending to do things in groups rather than attending classes individually. Another expressed the view that the proposed study might be “a tougher sell” (interview 3) to this population because of the cultural traits of this group. In particular, she cited tendencies to be very private about personal events such as stillbirth and many African Americans rely more on religious faith than on other external sources of help. She explained:

*… We’re not a community of people culturally that lean on outside help … So I don’t know how easy it is going to be to get people to say that this is what happened to me and so now I'm going to go to this activity to try and get better. Black people go to church … When there’s a problem if they are faith-based they will go to church and they leave it on their altar and they don't talk about it anymore (interview 3).*

The other African American participant expressed similar views and highlighted in particular the stigma perceived to be associated with any form of “mental unwellness” in ethnic minority groups generally. She indicated that this cultural attitude might present a barrier to women who had experienced stillbirth but would resist anything perceived as support offered to help them cope with their experience:

*I think (among) minorities in general there's a stigma attached to … any sort of mental unwellness … You know when I was in the hospital and they give me this folder full of resources … and I remember looking at the listings for the support groups and thinking I would never sit in a room and talk about this … When people think yoga - I have had people say “Oh that's for white people” (interview 5).*

One of the Hispanic participants expressed a similar view that yoga or similar healthy lifestyle strategies are not a strong focus of Hispanic culture in general:

*… I don’t wanna sound horrible… yoga just isn’t very popular here in this area for Hispanic women.*

#### Preferred yoga environment

The interviews explored participants’ views on the type of yoga environment that would be preferred by themselves or by other women in their ethnic group that had experienced a stillbirth. When asked for their views on the option of online yoga at home, all of the participants indicated that they would be receptive to this, either because of the perceived benefits of convenience or privacy or because of the constraints they currently face on attending classes outside the home.

Three of the participants mentioned that they would feel more comfortable or less self-conscious about taking part in yoga at home rather than in a class or suspected that other women would feel the same way.

For two of the participants, the preference for participating in yoga at home rather than in a class related to their unease about going out and interacting with other people so soon after their stillbirth experience:

*For me personally, anything at home would be better cause there is just, I don’t want to be, I don’t want to go out in public sometimes … and be around a lot of people (Interview 4).*

*Sometimes … I just don’t want to leave the house. I can’t leave the house. I just don’t want to deal with the outside world. I don’t want to go to a grocery store and hear a crying baby or you know have run into somebody who hasn’t seen me since I was pregnant and asks me where is the baby or how is the baby, so sometimes I just want to be at home and I don’t have to deal with any of that anxiety …. I can just do it when I’m ready … (interview 5).*

Time flexibility was cited as another benefit of participating in online yoga at home, with one participant mentioning the possibility of doing so at night when unable to sleep and another indicating that, although she might prefer to attend a yoga class in person, her current childcare responsibilities would make an online class easier (interview 5).

Two of the African American participants expressed the view that other Black women would be more likely to take part in yoga at home, with one stressing the greater ease of access to this form of participation in ethnic communities where yoga studios are rarely found, and the other noting that they might feel less comfortable in classes which tend to be dominated by White women. This participant indicated that an online program done at home might be more attractive to this population, especially if a social option was included such as the option of participating with a friend:

*Especially those African American and minority women who are living in communities where those studios and … options for private and/or group instruction are [not] available … I mean we are living in a Netflix generation where people just like to click on the television and everything is kind of being brought to them …. that would probably be the best way to lure them in (interview 3).*

*They wouldn’t have to feel awkward being in a room of all Caucasian if they want they can have friend over (interview 1).*

### Suggested recruitment methods

The main recommended recruitment channel was social media. One of the main themes that emerged in this area was the value of using referrals from trusted sources of information, such as medical professionals, counselors, or websites/magazines popular with the relevant ethnic minority population. Four of the five participants specifically recommended the use of social media, such as Facebook and Twitter, for recruiting women in their ethnic group.

In explaining why she felt social media would be a good way of connecting with individuals who had experienced stillbirth, one African American participant mentioned how valuable this had been to her as a means of support, in the form of virtual communications with women from all over the world who shared a similar experience:

*I’ve actually interacted with (people from) all over the world because of social media and some of them have become really good friends just because we talk and they share their experiences and then I share mine and it’s a community (interview 5).*

Specific ways of using social media that were suggested by the participants included the use of a video or online testimonial, or contacting women via existing social media support groups:

*I think that having like a video …. almost like an ad campaign … showing a diverse group and maybe even video testimonials or sharing little blurbs of people that have done it ... (interview 5).*

It was also suggested that individuals might be encouraged to take part in an online support group in which they could share their experiences of the program with other participants, and also make new friends. Other forms of media recruitment that were suggested included advertising in newspapers or on television, or via the websites or publications commonly read by ethnic minority women:

*There are really popular publications that African American women read and they are trusted sources … like* essence.com*,* madamnoire.com *… so if they see something there they are going to be more inclined to at least click it on and look into it (interview 3).*

*One suggested “word of mouth,” which is seen as being “very powerful” (interview 1)*.

Interviewee 3 also explained that as a new mom, she receives many email invitations from different sources and only considers these if they are seen as legitimate and preferably from a trusted source. For example, the request to be interviewed for this study had been from her grief counselor.

Another of the participants also recommended that trusted healthcare professionals or counselors should be used to recruit potential participants but stressed the importance of ensuring that these professionals are actually familiar with the program and its requirements and have a good knowledge of it to pass on to their patients or clients. One also expressed a desire to have information about this type of study communicated by her healthcare professional, but commented that they did not have this role at present:

*I could love it if it was through my doctor’s office, but my doctor’s office has no clue what’s going on (interview 4).*

A point raised by one of the African American participants was that some individuals from ethnic minority groups may resent being targeted for participation in a study because of their ethnicity. She suggested that it would be better to target women in general for the study, and later screen them for ethnicity (interview 3).

The five interviewees were also asked for their views on the most appropriate time period after the loss to approach women about the yoga study. There was considerable variation in their responses, ranging from within a week to no time limit, and each participant gave a different answer to this question. One participant expressed the view that after 3 to 4 months would be the best time to recruit women, as mothers are very busy in the first few months with things that need to be done after the loss of their baby, and they are grieving too intensely at this stage:

*I had to give birth to her and then I had what am I going to do next? Funeral arrangements. Do I cremate her do I want to have a funeral? Then I have people coming and going. Then I have my doctor’s appointments … So I would say just wait to get calm but three to four months would be best (interview 1).*

Because the length of recovery from stillbirth time varies so greatly, one participant stressed that women should be made aware of the study early on, so that they can decide to participate whenever they personally feel ready to do so:

*I don’t think they are going to want to participate in yoga right away, but I would put it on their radar, so that when you are physically in a better place you can start to do something (interview 5).*

Also acknowledging the importance of personal readiness to participate in such a study, the participant from interview three suggested that some women might not feel ready until after they have a living child, and that in general, it would be best to wait at least a year. The participant who recommended trying to recruit women within a week explained this in terms of her personal experience of wanting to seek help and support at that point, but also stressed that it is never too late to approach and offer support to women who have experienced the death of a baby:

*For me personally that is when I have been most motivated to try to find help or answers or anything...while it’s still fresh on my mind … But … no matter how long it was … I don’t think it’s ever to late. I don’t think anybody ever feels like they’re not still challenged with dealing with it (interview 4).*

### Content of recruitment material

The participants were asked for their views on the preferred style and content of messaging material used to recruit individuals to the yoga study. Their comments related to both visual aspects and the wording of the recruitment material. With regard to the visual aspects, one participant recommended that there should be no images of children or pregnant women in the recruitment material (interview 1). Instead, the same participant suggested that the target population would relate more to a photo of a woman crying or looking sad, or alternatively, a yoga related image or symbolic image of a sun rising with connotations of a new beginning. Another participant recommended that in order to attract ethnic minority women to the study, it would be important to ensure that any recruitment material included images of an ethnically diverse group that they could relate to and not just white women:

*I think that you want somebody to really connect with. You know – oh, she looks like me, she’s like me … You just feel a sense of similarity I think (interview 5).*

In terms of the wording of recruitment material, one participant stressed that this should be empathetic, demonstrating an understanding of what women who have experienced a stillbirth are enduring (interview 1). Another of the participants stressed that the wording of any recruitment literature should be sensitive in acknowledging the suffering that women who have had a stillbirth continue to experience and not to suggest that this is something that can easily be overcome:

*I would not want to see anything about moving on ... or getting over … there’s no getting over this, you have to live with it … I think that the notion has to be that “This is new. This hurts. This sucks. This is the worst possible situation and nothing is going to make you feel better, but this might make you feel a little less bad” (interview 5).*

The same participant also suggested, however, that it might be worth carefully including reference to the physical health benefits offered by yoga, which some women might respond positively to once they reach a certain stage after the loss of their baby.

### Incentives for participation

All of the participants agreed that it would be appropriate and effective to offer women a small monetary reward to compensate for their participation in the proposed yoga study. Overall, the main view seemed to be that this should be between $20 and $50, or the equivalent amount on a gift card redeemable at a store of their choice. Other suggestions included a gift basket or a yoga video disk that they could continue using at home after completion of the study.

Finally, one participant suggested that incentives designed to honor the child might encourage participation:

*I think that something that acknowledges their children might help, maybe giving them the choice of some kind of pendant or a card with their baby’s name … printed on it or something … Or even it were to be like to be a $10 donation to the charity of their choice on their behalf, in honor of their baby. … if you could customize something that would be great or if you could give them something physical that they can hold and say this reminds me of my baby (interview 5).*

### Revisions to recruitment flier

**Fig. 3:**
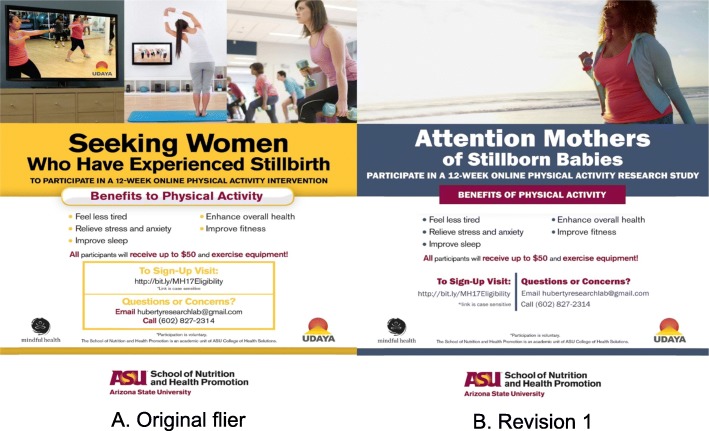
Revisions to the flier

Based on the aforementioned recommended strategies to recruit minority women into a research study for online yoga, several revisions were made to the original recruitment flier. See Fig. 3 for the revisions.

## Discussion

The purpose of this paper was to present findings from phase one (i.e., iterative design) of a two-phase randomized feasibility study. The iterative design included (1) the development of online videos used in phase two (i.e., online yoga intervention currently in progress) and (2) phone interviews to determine perceptions of yoga and preferred recruitment strategies of minority women.

### Development of online yoga videos

We found that the majority of the tested online videos (*N* = 25/30) increased self-compassion (SC) and/or emotional regulation (ER) to some degree immediately after engaging in a yoga video. We do not know whether this increase persisted beyond the completion of the video, and it is important to note that we used an investigator-developed 1-item Likert scale (not validated) to reduce participant burden that may have occurred by completing a validated measure for SC (26 items) or ER (10 items) before and after engaging in a yoga video. There is literature to suggest that SC [39, 40] and ER [41] may be important factors in coping with symptoms of PTS. Common symptoms of PTS include recurring flashbacks, hyper-vigilance, hyper-arousal, and experiential avoidance [42]. Yoga’s effectiveness may be partially attributed to improvements in SC and ER [40, 43–45]. Specifically, yoga may increase affect tolerance, attention, and awareness which may help increase ER and potentially reduce avoidance and re-experiencing symptoms [39, 40, 46]. We are currently testing the overall SC and ER scores (using validated questionnaires) in phase-two of the study.

In addition to testing the pre-selected online yoga videos, we developed yoga videos utilizing population sensitive language (i.e., speaking to grief, loss, and mental health, no mention of babies) and awareness of physical limitations of women who experience stillbirth (e.g., minimal hip opening poses). According to the literature, tailored messaging (i.e., language), compared with non-tailored messaging, in health behavior change programs commands greater attention, are processed more intently, and may be perceived more positively by the individuals [47]. With that in mind, we developed a set of 12 online yoga videos that we believe women who experience stillbirth will be able to relate and connect with. Though not a goal of phase one (study reported here), we will explore women’s experiences of the online yoga intervention via qualitative interviews at the conclusion of the study in phase two. Findings will be published at that time.

### Interviews

We conducted interviews in five bereaved mothers who identified themselves as either Black, African American, or Native African and/or Hispanic and the findings (discussed below) may inform future, more inclusive recruitment efforts for complementary approaches, such as yoga, in addition to the recruitment for phase two (currently ongoing).

#### Perceived benefits and barriers

Minority women perceived physical and mental/psychological benefits of yoga participation specific to helping them to cope with their emotions (e.g., sadness). Spadola and colleagues [48] also reported that racially ethnic diverse adults perceive yoga to have positive effects on physical and mental health and overall well-being. The barriers shared by participants were not different from what is reported in pregnant women and women who have experienced live birth [49] and included cost, time, and lack of energy. Three of the five women reported costs as potential barriers for participation. Others have reported yoga is perceived as more expensive than other forms of mindfulness practice and/or physical activity [50].

#### Perceived interest in yoga

Although the sample size is small (*N* = 5), there was interesting and useful information shared in the interviews related to the perceived interest in yoga. Four of the five women did not think that participation in a yoga study would be of high interest in other women who had also experienced stillbirth and/or were of ethnic minority. This was mainly due to cultural traits. For example, African American women rely on their spiritual practice (i.e., religion) rather than other sources for support, and this has been reported in other studies [51–53]. Conversely, one study conducted in diverse and underserved/underprivileged populations (e.g., minority groups, elderly, gay populations) reported high interest in yoga [54]. Researchers implemented a 1-h yoga class, once per week, for 8–21 weeks across multiple sites. Nearly the entire sample (98%; *N* = 215/219) reported that they would recommend the yoga class to others. Fifty-seven percent of participants reported feeling a great deal of overall wellness from the yoga class, and 58% reported they were moderately likely to use the yoga postures in their daily life. Though this study included a more diverse sample of participants, it highlights the potential interest of minority and underserved/underprivileged populations.

Only one woman (Hispanic) thought that ethnic minorities would be interested in online yoga but suggested that women of all ethnic backgrounds would benefit from it. One African American woman shared that some individuals from ethnic minority groups may resent being targeted for participation in a study because of their ethnicity. Instead, she suggested targeting women in general for the study. This is an interesting finding because much of the literature related to recruitment and enrollment strategies suggest that participants may be more likely to respond to advertisements with ethnically specific statements compared with more general statements [55, 56]. However, women and minorities have been and continue to be underrepresented in clinical trials [57]. Additionally, stillbirth is more prevalent in certain ethnic groups, particularly African American women [4, 58].

There was also a suggestion that there could be stigma more generally in ethnic minority groups about having issues with “mental unwellness”, and this would be a barrier to participation to yoga. A systematic review investigating the impact of mental health-related stigma on help seeking reported that minority ethnic groups experience a mental health stigma that deters them from help-seeking [59]. Ethnic minority groups suffer double stigma when experiencing poor mental health as well as prejudice and discrimination, and this combination can prevent these individuals from seeking treatment [60]. Future studies should design more culturally relevant interventions to gain more interest and participation from ethnic minority groups. Also, one Hispanic woman suggested that culturally healthy lifestyles are not a strong focus so that something like yoga would not be a typical coping mechanism. Future studies could use more inclusive messaging to attempt to address the potential cultural barriers and associated stigmas related to yoga participation.

#### Preferred yoga environment

Women in our study who were interviewed were receptive to online yoga based on convenience, privacy, and other barriers commonly reported in interventions outside of the home. Some women mentioned feeling less self-conscious if practicing yoga at home (i.e., would not need to go out in public to attend a class). These experiences are not different from a small sample of predominately White women who experienced stillbirth and reported that they especially enjoyed the convenience and privacy of participating in yoga at their home [27]. One participant from this study indicated that online yoga at home might be even more attractive to the ethnic minority population, especially if a social option was included such as the option of participating with a friend. Women could easily invite trusted others into their home to practice yoga with them (provided they have the space to accommodate two people). Participation in yoga in minority populations is underrepresented because of the aforementioned views and due to perceived high costs, beliefs that yoga is mainly practiced by white, thin females, and lack of ability/self-efficacy to perform the poses [48, 61, 62]. Offering yoga in a different setting, such as online where participants can practice in the comfort of their home or even with a friend, might be a more attractive way to engage in yoga for ethnic minority women who have experienced stillbirth.

#### Suggested recruitment methods, content of recruitment material, and incentives

Overall, the responses suggested that it might be most appropriate to attempt recruitment of women several months after their stillbirth experience, but to do so in ways that acknowledge the fact that some women will not be mentally or physically ready to participate at that stage, and also not to rule out the possibility that some women may be receptive to the invitation much earlier. Women also expressed a perceived importance of “trusted sources” in recruitment of women from ethnic minority groups. The findings relating to the style and content of recruitment material help to inform how this might be done and are listed below:

- There should be no images of children or of pregnant women in the recruitment material. Instead, include a photo of a woman crying or looking sad, or alternatively a yoga-related image or symbolic image of a sun rising, with connotations of a new beginning.
- Recruitment materials should also include images of an ethnically diverse group with whom they could relate and not just images of white women.
- The wording of recruitment material should be empathic and sensitive to demonstrate an understanding of what women who have experienced a stillbirth are enduring. It is critical not to imply that women should “move on” or that stillbirth is a tragedy to be overcome.
- Carefully include reference to the physical health benefits offered by yoga, which some women might respond positively to once they reach a certain stage after the loss of their baby.
- Small monetary compensation for participation in an online yoga intervention would be appropriate, specifically between $20 and 50 or in the form of a gift card redeemable at a store of their choice.
- Gift baskets or a yoga video disk that they could continue using at home after completion of the study may also be acceptable compensation.
- Incentives designed to honor the child might encourage participation (e.g., donation to charity, pendant, card).

### Limitations

The findings from phase one of this study provide important insights to be applied to phase two of this NIH funded trial, but several limitations should be noted. First, the sample size is small and may not reflect the wide range of experiences and attitudes of all minority women who have experienced stillbirth. However, due to the purpose of the study (i.e., inform development, inform recruitment), this sample is acceptable [63]. To our knowledge, no studies of this nature have been conducted in minority women who have experienced stillbirth, and as such, this data contributes to the limited evidence base about their perceptions of yoga and preferred recruitment strategies of this population. Future studies should consider larger samples. Second, we did not use a validated questionnaire to assess emotion regulation or self-compassion pre-post video review but rather a 1-item Likert scale question to reduce participant burden and to get a preliminary sense of the types of online videos to be used in phase two. The full-length validated scales will be used in phase two of the trial. Third, we relied on self-report data which may be subject to social desirability, response, or recall bias. Fourth, the majority of participants in this study were White. While there is no literature to suggest that White women may have differing responses regarding emotion regulation and self-compassion after watching an online yoga video, future studies should consider demographics. This includes making an effort to recruit racial minorities as well as having a diverse representation of teachers and students in the videos. Finally, women who reviewed the online yoga videos did so in a laboratory setting or at home via web-conferencing. It is unknown how the environment in which the participant practiced yoga might have influenced their emotion regulation or self-compassion scores.

## Conclusions

These findings informed the development and testing of an online yoga feasibility study via use of an iterative design centered on input from ethnic minority women who have experienced stillbirth. For example, our recruitment materials used empathetic language and pictures of ethnically diverse women. For advertising, we reached out to stillbirth support groups as well as groups that cater to an ethnically diverse clientele of women. We also provided monetary incentive to compensate for the time spent in the study and sent emails to recognize the birthday of the mother’s stillborn child. Developing videos that include racial/ethnic diversity of teachers and students and testing the online yoga videos via an iterative design adds strength to this study. If proven effective (currently being tested in phase two), these videos could be used for other research studies and/or disseminated more widely to women after stillbirth. Additionally, ethnic minority women are receptive to participation in online yoga to cope with symptoms of PTS after experiencing stillbirth. Many also perceive online yoga to be beneficial but express that recruiting ethnic minority women needs to be specific. Future research should consider adapting culturally sensitive language in recruitment materials in order to gain interest of ethnic minority women who have experienced stillbirth. Alternative ways of coping with PTS after stillbirth are sorely needed, especially given that women desire nonpharmacological, home-based approaches in this sensitive time period. Online yoga is a novel, low-risk approach to reduce symptoms of PTS, and the home-based nature may improve uptake and acceptance in future interventions. Although findings are based on a small, convenience sample, they may help to inform future efforts to recruit ethnic minority women for yoga interventions, especially for those who have symptoms of PTS following stillbirth.

## Data Availability

Data and materials are available from the corresponding author on reasonable request.

## Abbreviations

ER: Emotion regulation
PTS: Post-traumatic stress
SC: Self-compassion
SPSS: Statistical Package for the Social Sciences
